# Novel Bacterial Taxa in the Human Microbiome

**DOI:** 10.1371/journal.pone.0035294

**Published:** 2012-06-13

**Authors:** Kristine M. Wylie, Rebecca M. Truty, Thomas J. Sharpton, Kathie A. Mihindukulasuriya, Yanjiao Zhou, Hongyu Gao, Erica Sodergren, George M. Weinstock, Katherine S. Pollard

**Affiliations:** 1 The Genome Institute, Washington University School of Medicine, St. Louis, Missouri, United States of America; 2 Gladstone Institutes, University of California San Francisco, San Francisco, California, United States of America; 3 Division of Biostatistics, Institute for Human Genetics, University of California San Francisco, San Francisco, California, United States of America; Baylor College of Medicine, United States of America

## Abstract

The human gut harbors thousands of bacterial taxa. A profusion of metagenomic sequence data has been generated from human stool samples in the last few years, raising the question of whether more taxa remain to be identified. We assessed metagenomic data generated by the Human Microbiome Project Consortium to determine if novel taxa remain to be discovered in stool samples from healthy individuals. To do this, we established a rigorous bioinformatics pipeline that uses sequence data from multiple platforms (Illumina GAIIX and Roche 454 FLX Titanium) and approaches (whole-genome shotgun and 16S rDNA amplicons) to validate novel taxa. We applied this approach to stool samples from 11 healthy subjects collected as part of the Human Microbiome Project. We discovered several low-abundance, novel bacterial taxa, which span three major phyla in the bacterial tree of life. We determined that these taxa are present in a larger set of Human Microbiome Project subjects and are found in two sampling sites (Houston and St. Louis). We show that the number of false-positive novel sequences (primarily chimeric sequences) would have been two orders of magnitude higher than the true number of novel taxa without validation using multiple datasets, highlighting the importance of establishing rigorous standards for the identification of novel taxa in metagenomic data. The majority of novel sequences are related to the recently discovered genus *Barnesiella*, further encouraging efforts to characterize the members of this genus and to study their roles in the microbial communities of the gut. A better understanding of the effects of less-abundant bacteria is important as we seek to understand the complex gut microbiome in healthy individuals and link changes in the microbiome to disease.

## Introduction

The human gut harbors thousands of bacterial taxa [1], a small number of which dominate the community [1], [2]. Early studies using sequence-based assays indicated that the majority (62–76%) of taxa in the gut microbial community are uncharacterized [3], [4]. However, many new taxa have been identified in the profusion of metagenomic sequence data subsequently generated from human stool samples [2], [5]–[7], raising the question of whether more taxa remain to be identified. The large number of taxa that occur with low abundance [1] (Zhou et al, manuscript in preparation) suggests that there may indeed be rare taxa that remain unobserved at current sampling depths.

We assessed metagenomic data generated by the Human Microbiome Project Consortium to determine if novel taxa remain to be discovered in stool samples from healthy individuals. Characterizing the full complement of taxa in the human gut is an important step toward understanding the role of the gut microbiome in human health and disease. To do this, we established a rigorous sequencing and bioinformatics pipeline with sequence data from multiple platforms (Illumina GAIIX and Roche 454 FLX Titanium) and approaches (whole-genome shotgun (WGS) and 16S rDNA amplicons). We applied these techniques to stool samples from 11 healthy subjects collected as part of the Human Microbiome Project (HMP) [8]. We found several low-abundance taxa confirmed by multiple datasets.

## Results

### Datasets and Criteria for Identifying Novel Sequences

Four distinct datasets were used in this analysis (Table 1, Tables S1 and S2; Figure S1). The first three datasets were derived by examining stool samples from 8–11 healthy individuals and used to identify potentially novel sequences. The first and second datasets (Figure S1A and Table S1) consist of Roche 454 FLX Titanium sequences from 16S rDNA amplicons from variable regions 1–3 (Roche V1–3) and 3–5 (Roche V3–5). Together the sets are referred to as the “Roche variable regions” data. The third dataset consists of 16S rDNA sequences identified from randomly sheared, metagenomic DNA fragments that were shotgun-sequenced on the Roche platform (Roche WGS) (Figure S1B and Table S1). The final dataset was derived from stool samples from 94 individuals and was used to validate the potentially novel sequences. These data consist of 16S rDNA sequences generated from both ends of randomly sheared, metagenomic DNA fragments that were shotgun sequenced on the Illumina platform and identified as 16S rDNA by alignment to 16S rDNA reference sets (Illumina WGS) (Figure S1C and Table S2).

**Table 1:** Sequence statistics.

	Use	Subjects	Samples	Read length before trimming (Roche lengths are approximate)	Total	Non-redundant 16S reads	Non-chimeric (ChimeraSlayer)	<97% identity to known 16S	Illumina support	Illumina tiling pattern	Illumina spanning pair	Novel PhylOTU OTUs
Roche V1–3	Identify	8	12	200–500 bp	92,330	67,789	54,599	4,963	1,838	128	30	7
Roche V3–5	Identify	11	20	200–500 bp	160,948	125,106	106,934	5,210	1,388	59	26	3
Roche WGS	Identify	11	12	50–600 bp	33,236,142	52,756	n/a*	336	115	85	85	21
Illumina WGS	Validate	94	147	100 bp paired-end	67,679,702 (16S reads)	n/a	n/a	n/a	n/a	n/a	n/a	n/a

* not applicable.

We defined novel Roche V1–3, V3–5, and WGS reads as those that met the following criteria: (1) less than 97% identity to known 16S rDNA sequences in the NCBI NT and the Ribosomal Database Project (RDP) databases [9], [10], and (2) confirmed by Illumina WGS data, as described below, to ensure that the novel 16S rDNA sequences were not the result of platform bias, sequencing error or PCR artifact.

**Figure 1:**
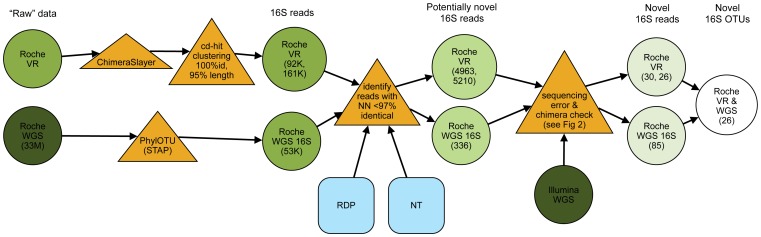
Flow chart describing the pipeline for the identification of novel OTUs. Green circles represent the data collected by the HMP, with the darkest green circles representing WGS read, and successively lighter green circles associated with 16S reads that show progressively more evidence of novelty. Blue squares represent external databases used to determine novelty. Major filtering steps in the pipeline are shown as yellow triangles.

Our pipeline for analysis of novel 16S rDNA bacterial sequences is shown in Figure 1. Identifying novel reads began with running Roche variable region sequences through Chimera Slayer [11] and clustering non-chimeric reads at 100% identity to remove duplicate sequences. 16S rDNA sequences were identified from the full Roche WGS dataset with PhylOTU [12]. Next, those reads with <97% identity to a nearest neighbor in the NCBI NT and RDP databases were considered potentially novel (see Methods). Based on this threshold, 4–7% of non-redundant sequences (4,963 Roche V1–3 reads and 5,210 Roche V3–5 reads) and about 0.6% (336) of Roche WGS reads identified as 16S rDNA were potentially novel (Table 1). Finally, we aligned the Illumina WGS data to the potentially novel Roche WGS and variable region sequences and used 100% identical alignments to validate the Roche sequences. The distribution of Illumina WGS reads across the length of each Roche read was used to identify sequencing errors and chimeras in the Roche data, as described in detail below. High-quality Roche WGS and variable region sequences with Illumina WGS support were considered candidates for truly novel taxa.

### Identifying Novel 16S rDNA Sequences with Data from Two Sequencing Platforms

Our goal was to identify novel 16S rDNA sequences in the Roche datasets that were strongly supported by Illumina WGS sequence data. Given that Roche sequences could be misidentified as novel because of sequencing error, chimerism (despite having been screened for chimeras, Figure 1), or real variability from previously identified bacterial taxa, we distinguished Roche sequences with real variability from known taxa by analyzing the alignments of Illumina WGS sequences to the Roche sequences as follows.

We excluded any Roche variable region or WGS sequences that were not supported by Illumina WGS reads with perfect alignments over at least 95% of the length of the Roche sequence (see Methods for details). We opted for this stringent criterion so as to identify only the highest confidence reads lacking sequencing errors that could result in an over-estimation of the number of novel 16S rDNA sequences. Very rare Roche variable region and WGS reads might not have been represented in the Illumina WGS data, and thus were not supported. Application of these criteria allowed us to narrow our focus to less than 2000 candidate novel reads (1–3% of non-redundant sequences) for each Roche variable region dataset and 115 reads (0.2%) for the Roche WGS data (Table 1).

**Figure 2:**
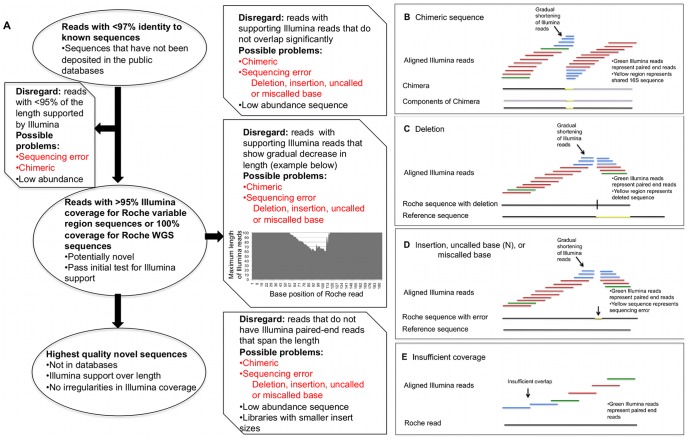
Irregularities in Illumina coverage used to identify chimeras and sequencing error. (A) This flow chart describes the removal of Roche WGS and variable region sequences based on characteristics of the Illumina alignments, resulting in a final set of high-quality novel sequences. Possible problems listed in red indicate errors in the Roche sequence, and possible problems listed in black indicate situations in which the Roche read may be of high quality but could not be confirmed by aligned Illumina sequences. The inset plot shows an example of a Roche variable region sequence with a progressive shortening of aligned Illumina sequences. The base position of the Roche sequence at which the Illumina alignment begins is plotted on the x-axis, and the maximum length of aligned Illumina reads with the given start position is plotted on the y-axis. In the middle of the Roche sequence, the Illumina sequences decrease to approximately 60 bp before returning to 100 bp in length. Characteristics of the Illumina alignments associated with specific problems with the Roche WGS and variable region reads are illustrated in B–D.

**Figure 3:**
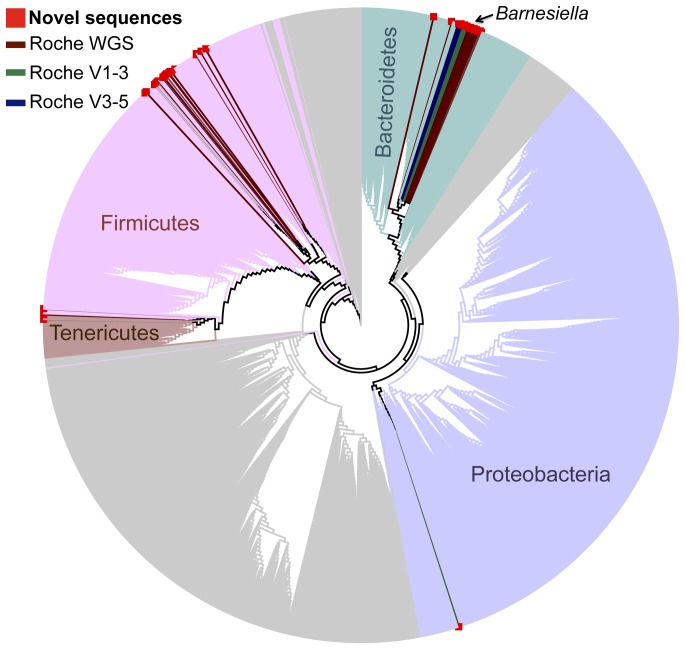
Taxonomic distribution of novel reads. Novel reads from each dataset (dark colored branches, see legend) were inserted into the Silva reference tree with RAxML and are marked with a red box at the end of the leaf. Novel reads cluster into phyla that are typically found in stool samples (light colored branches, as labeled); all remaining phyla are shown in gray. The black arrow indicates the group of reads identified as the genus *Barnesiella*.

The alignment patterns of the Illumina WGS sequences to the Roche data distinguished high-confidence, novel Roche WGS and variable region sequences from chimeric sequences (Figure 2B), deletions (Figure 2C) and insertions, uncalled bases or miscalled bases (Figure 2D). We also removed sequences that did not have sufficient overlap of aligned Illumina sequences to confidently confirm the Roche sequence (Figure 2E). These irregular Roche sequences were revealed by three characteristics of the Illumina alignments: (1) the maximum length of the Illumina sequences that align starting at each position within the Roche sequence gradually decreases and then increases (Figures 2B–D), (2) Illumina sequences did not overlap each other significantly (Figures 2B–E), and (3) Illumina paired-end sequences did not span ≥90% of the length of the Roche sequence (Figure 2B–E). These Illumina alignment patterns are described in detail in the Methods and are graphically depicted in Figure 2. In the Roche variable region datasets, these irregularities served as a database-independent method for detecting chimeric sequences (Figure 2B), and indeed, the majority of sequence irregularities in Illumina coverage indicated the presence of a chimeric sequence. Strikingly, this analysis of the Illumina tiling patterns reduced the number of high-confidence, novel sequences from about 2000 candidates to 30 high-quality novel Roche V1–3 and 26 Roche V3–5 sequences in each dataset (Table 1). In the Roche WGS dataset no chimeric sequences were found, and the reduction in the number of novel sequences was less severe, resulting in 85 high-quality novel Roche WGS sequences. Descriptive information about the novel sequences from each Roche dataset, including RDP classification and nearest-neighbor descriptions based on alignment, is shown in Table S3.

### Novel Taxa Found in Amplicon and WGS Datasets Partially Overlap

The taxonomic distribution of the novel sequences generally reflects the overall taxonomic structure of stool samples: novel Firmicutes, Bacteroidetes, and Proteobacteria were identified (Figure 3). A single read was poorly identified as Actinobacteria by RDP (bootstrap value of 0.31); the absence of additional novel taxa within Actinobacteria may simply reflect random sampling of the very-low-abundance novel taxa.

Several methods were used to compare the novel reads found from the Roche variable region and WGS datasets. Reads were taxonomically classified down to the genus level by the RDP classifier (version 2.2 with training set 6) (Table S3), inserted into the Silva reference tree (Figure 3), and clustered into operational taxonomic units (OTUs) by PhylOTU [12](Table S3). The WGS reads were then aligned against the variable region reads using NCBI BLAST [13] (details provided in the Text S1).

Overall, the methods agreed (see Text S1 for an explanation of differences). A large percentage (56%–96%) of novel reads from each of the three datasets were identified by the RDP classifier as belonging to the genus *Barnesiella*, shared significant sequence identity, clustered into OTUs 12 and 13, and were placed together on the Silva tree. OTUs 7 and 11, both identified as belonging to the order Clostridiales, also contain reads from both the Roche WGS dataset and one of the Roche variable region datasets. The remaining sequences were distributed across the major bacterial phyla typically found in stool samples and produced OTUs with one sequence (OTUs 1–3, 5, 6, 8–10, 14–22), two sequences (OTUs 4,23, 26), or four sequences (OTUs 24 and 25) from a single dataset (Table S3).

### The Most Abundant Novel Taxa Belong to the Genus *Barnesiella*

The majority of novel sequences in each dataset were assigned to the genus *Barnesiella* (Figure 3, indicated by arrow). In particular, the two largest OTUs (OTUs 12 and 13) contained reads identified as *Barnesiella* or identified with low confidence as a different genus within the same family or order as *Barnesiella*. These OTUs have substantial representation from the Roche WGS data and at least one Roche variable-region dataset, further validating the observations of these taxa and indicating that they are sufficiently abundant to be reliably detected using different sequencing methods. Nonetheless, the sequences belonging to this OTU included, at most, 1.3% of the total sequences generated from a single sample. This abundance is comparable to a rare organism, such as *Escherichia coli*, which comprised a maximum of 0.94% of the sequences in stool samples from healthy HMP subjects (The Human Microbiome Consortium (2012) ‘Structure, Function and Diversity of Human Microbiome in an Adult Reference Population’. Nature: doi:10.1038/nature11234)(dataset: hmp1.v13.hq.otu.counts, OTU263 classified as Escherichia/Shigella by RDP). Interestingly, the sequences in these two OTUs are predominantly from a single subject. Both OTUs have support from multiple visits, indicating that these novel taxa are persistently present in this individual’s stool. Both OTUs have percent identities to nearest neighbors and RDP classifications indicating that these taxa are not vastly divergent from previously observed taxa. Alignments to cultured *Barnesiella* are <90% identical, indicating that we may have identified novel *Barnesiella* species or a closely related genus.

### Novel Taxa Largely Represent Novelty at the Species Level

**Figure 4:**
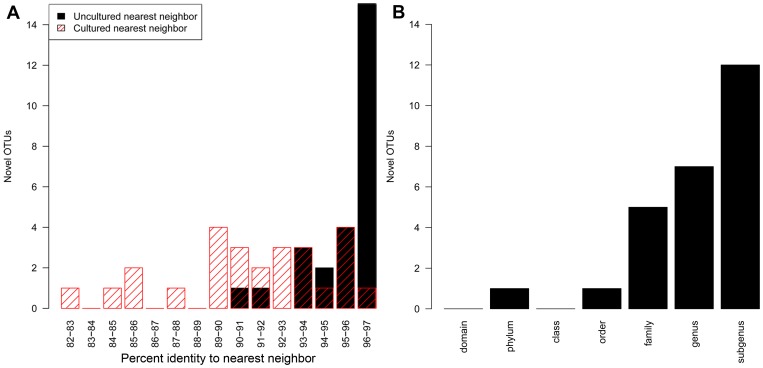
Novelty at different taxonomic levels. (A) Distribution of novel OTUs as a function of the maximum percent identity of a constituent read to a nearest neighbor for (black) all reads in the RDP and NT databases and (red) cultured reads. (B) The distribution of novel OTUs as a function of the lowest taxonomic rank that was confidently (with a bootstrap value of >0.5) assigned to a constituent read.

Novel taxa in human stool samples are not expected to be very divergent from known taxa because previous studies, particularly of the 16S rRNA locus, have already captured the major phyla and classes of gut bacteria. As expected, RDP classifies the majority of OTUs with confidence at least to an order and nearly half to a genus (Figure 4B). The two OTUs poorly classified by RDP at the phylum and order level are examples of reads that are particularly difficult to classify (since each OTU contains a single sequence which is less than 200 bp long) and probably do not represent novelty at a high taxonomic level. Similarly, most novel OTUs have relatively high percent identity to previously sequenced 16S rDNA (Figure 4A). We also identified the nearest neighbor within the set of cultured bacteria (see Methods) to further assess how divergent the novel taxa are from cultured, well-characterized bacteria strains. All but two novel sequences were most similar to uncultured bacteria, and the percent identity to a nearest neighbor from cultured bacteria is significantly lower than from uncultured bacteria, on average 4% lower (Figure 4A). The RDP classification and percent identity to nearest neighbors indicate that the novel taxa observed are primarily from genera that have previously been sampled. We observed only a few sequences that may be novel at the taxonomic level of family or higher. The most novel OTU by percent identity contains a single sequence, which has 90.5% identity to its nearest neighbor, but is still well classified by RDP as the genus *Barnesiella* (Table S3). The next most novel OTU is perhaps a better candidate for novelty at family level or higher, with a single read which has 91.8% identity to a nearest neighbor, only 85.1% identity to a cultured nearest neighbor, and confident RDP classification only as low as the order Clostridiales. While new bacterial phyla may still exist, none was found in this dataset with our stringent filtering of reads.

### Novel Taxa are not Abundant but are Present in Multiple Individuals

Novel OTUs are generally of low abundance within individuals. In any one individual, no novel cluster comprised more than 1% of the total reads in the sample. This indicates that in stool samples, the abundant bacteria have already been sampled and deposited into the databases we queried.

**Figure 5:**
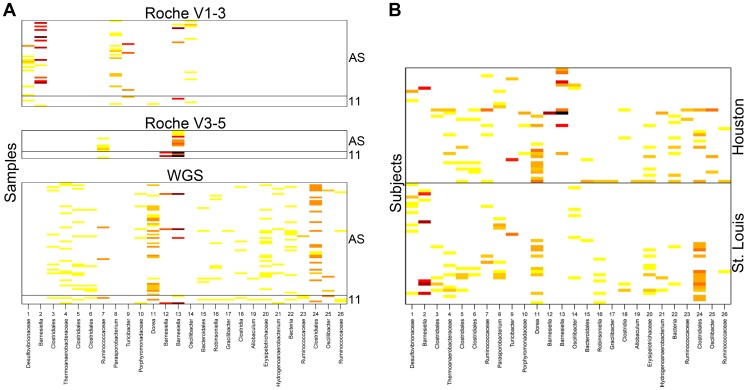
Distribution and abundance of novel OTUs. (A) The number of reads assigned to each OTU (x-axis) across all the samples (y-axis) is represented by color on a scale from light yellow (few sequences) to dark red (many sequences). Within each dataset (“Roche V1–3”, “Roche V3–5”, and “WGS”), samples are organized by their source: the three smaller groups of samples labeled “11” contain the original reads (from 11 subjects) used to define the OTUs. Reads recruited to the OTUs from the extended Roche variable region datasets (“Roche V3–5” and “Roche V1–3”) and Illumina WGS dataset (additional samples, “AS”) are also shown. (B) The total number of reads assigned to each OTU (x-axis) across all subjects (y-axis) is represented by color on the same scale. These data include the original reads as well as reads from both extended variable region datasets and the Roche and Illumina WGS datasets. The subjects in the lower portion of the figure were sampled in St. Louis while those in the upper portion of the figure were sampled in Houston.

To further assess the distribution and abundance of novel OTUs across a larger set of subjects, sequences from stool samples from additional healthy subjects collected for the HMP were screened for the presence of the novel OTUs (see Methods). Figure 5 shows the distribution of the 26 OTUs in the full set of available stool samples/subjects. The majority of OTUs are present in multiple subjects (Figure 5B), indicating that these novel taxa are widespread in the small population sampled. OTUs 17 and 19 are only present in one subject and are each composed of just one Roche WGS read, suggesting that they were not detected in additional individuals due to extremely low abundance. OTU 12 contains reads from both the Roche WGS and V3–5 datasets from multiple samples but only a single individual, indicating that this OTU is relatively more abundant (although still low) than the other OTUs, but specific to a single subject. Figure 5B is stratified by the sampling location of the subjects (St. Louis or Houston). Most OTUs are split relatively evenly between sampling locations. However, OTUs 10, 12 and 13 only appear in the Houston cohort and are all identified as the genus *Barnesiella*, which could mean these closely related taxa are specific to or more abundant in the communities sampled from this region because they are well suited to the gut environment in this region or population. OTUs 17, 19, 23, and 25 are also only found in the Houston cohort, but this is likely due to chance sampling of these very-low-abundance OTUs.

### Assessment of Primer Bias in the Detection of Novel Sequences

One reason the Roche WGS and Roche variable region datasets could differ in the taxa identified is because of the potential for primer bias in the variable region amplicons. To assess if novel taxa found only in the Roche WGS dataset (Figure 5A) were potentially detectable with variable region sequencing, the novel Roche WGS reads were aligned to the V1–3, V3–5 and V6–9 primer sequences using NCBI BLAST. Nearly every novel Roche WGS sequence that overlapped a priming site had 100% identity to a primer sequence (four sequences had one or two mismatches with respect to a primer but likely would still amplify, see Methods). Although the Roche WGS sequence did not span both primer sites, these data suggest that differences in the taxa found in the datasets were not due to primer bias, but rather to random sampling of very-low-abundance taxa. Primer bias may still limit the novelty observed, but the limitations may not be apparent due to insufficient sequencing depth or the effects of the 16S rDNA sequence identification and trimming steps applied to the Roche WGS data. In addition, biases in sampling, preparation, storage and DNA extraction could restrict the novelty observed in both the Roche variable region and WGS samples.

## Discussion

We discovered a number of new bacterial taxa, all but three of which were represented in multiple subjects from a larger set of stool samples from over 80 healthy individuals. These findings establish that bacterial taxa remain to be discovered, even in well-studied microbial communities such as the healthy human gut. We also showed that the number of false-positive novel sequences identified in the 11 samples would have been two orders of magnitude higher than the true number of novel taxa without validation by multiple datasets. For example, the discovery of novel bacterial taxa using metagenomic 16S rDNA sequence data was complicated by artifacts, such as sequencing errors and chimerism, which generated false signals of novelty. This study highlights the importance of establishing rigorous standards for the identification of novel taxa in metagenomic data. The pipeline we developed is an important first step towards establishing experimental and bioinformatics criteria for these standards.

Specifically, we found that leveraging Illumina WGS data to validate potentially novel Roche variable region and WGS 16S rDNA sequences enabled, with a high level of confidence, the identification of novel taxa in stool samples from healthy subjects. The use of independently derived datasets to confirm sequences allowed us to avoid over-estimating the fraction of novel sequences within the datasets; instead, we identified a small subset of sequences that warrant further investigation. Overall, we estimated that less than 0.07% of high-quality, non-redundant 16S rDNA sequences are truly novel, based on our strict definition of novel as sequences that are at most 97% identical to any sequence in public databases.

The false positives that we eliminated based on our rigorous validation requirements included sequences that appeared novel because of chimerism and sequencing errors. Of the 16S rDNA sequences with less than 97% identity to 16S rDNA reference sequences, less than 1% of the Roche variable region sequences were validated while 25% of the Roche WGS sequences were validated (Table 1). This discrepancy in the percent of reads validated in each dataset is due to the presence of chimeric sequences in the Roche variable regions data. The Roche variable region sequences had been screened for chimeras, but chimeric sequences were still abundant in the subset of sequences that appeared to be novel. Of the potentially novel sequences, the chimeric sequences were the most divergent from a nearest neighbor and were frequently classified poorly by RDP. Thus, an analysis of novel sequences without our validation steps would have grossly overestimated the number of novel sequences and the taxonomic level at which diversity was found. Sequencing errors (insertions, deletions, miscalled or uncalled bases) were also a source of false-positive novel sequences. We did not expect or detect chimeric sequences in the Roche WGS dataset, and the false-positive novel sequences in this case were due predominantly to sequencing errors. While sequencing errors can cause sequences to appear more divergent from a nearest neighbor, small errors do not affect RDP classification. It is likely that including sequences with sequencing errors would have slightly inflated our estimation of novel sequences, but the diversity still would have appeared at the genus or subgenus level. The artifacts we eliminated by using Illumina WGS sequences for validation allows us to more accurately define the degree of novelty and the characteristics of the novel taxa, including their distribution among individuals, abundance, and taxonomic classification.

Using the Illumina WGS data to evaluate the Roche variable region datasets establishes a database-independent method to detect chimeric sequences. Although the Roche V1–3 and V3–5 sequences had been screened with Chimera Slayer [11], some chimeric sequences were not identified because Chimera Slayer (and other database-dependent methods of chimera detection) only detects chimeras with components that are similar to the sequences in the reference database [11]. Chimeric sequences can be very abundant in 16S rDNA amplicon datasets, accounting for more than half of the diversity in OTUs in some samples [11], and the same chimera can be formed in independent amplifications of the same sample [11]. Chimeric 16S rDNA sequences are likely to be deposited in public databases at a relatively high rate [14]. Chimeric sequences confound 16S rDNA analyses that rely on accurate sequences for alignment, the generation of distance matrices and phylogenetic tree construction, and they also inflate the degree of novelty estimated in bacterial communities. Thus, novel methods of chimera detection, such as the one presented here, can improve the analysis of metagenomic data.

While several distinct clusters of reads were found across the bacterial tree of life, these sequences represent the diminishing portion of the human gut microbiome that remains unobserved. Earlier studies estimated that proportion was much higher. The novel taxa are of relatively low abundance (<1% of the total reads in a sample), but some are found in multiple individuals (0.5–20% of individuals at this depth of sequencing), and this recurrence suggests they are endemic to the human microbiome. Low-abundance organisms may have important roles in microbial communities. Dominant organisms in a community can be used to broadly define microbial habitats in the human body [15] (The Human Microbiome Consortium (2012) ‘Structure, Function and Diversity of Human Microbiome in an Adult Reference Population’. Nature: doi:10.1038/nature11234) and even microbial community classes within individuals [2] (Zhou et al, manuscript in preparation). However, even minor components of bacterial communities may influence human health [16] or can overgrow under certain conditions to cause disease [17]. The great diversity in these low-abundance organisms may allow us to further distinguish individuals by their bacterial communities or ascribe functional characteristics related to human health and disease.

We detected novel taxa in the three most prevalent phyla in stool samples: Bacteroidetes, Firmicutes, and Proteobacteria. Most of the diversity we detected was at the genus or sub-genus level, but a few novel 16S rDNA reads were divergent from all other observed sequences at a higher taxonomic level, potentially as high as the family level. The novel sequences identified here indicate that there are undiscovered bacterial taxa. However, the functional capabilities and other characteristics of these bacteria remain to be determined by whole-genome sequencing, isolation and culturing of the organisms, and by studying of the role of these bacteria within their communities. The novel 16S rDNA sequences can be used to target specific bacteria for whole-genome sequencing and will give us insight into what gene functions these uncharacterized organisms are contributing to the gut microbial community. Indeed, many of the novel OTUs are related to organisms that have already been identified by the HMP reference genome sequencing effort and are sequenced or in the process of being sequenced (http://www.hmpdacc-resources.org/hmp_catalog/main.cgi). For example, isolates from *Barnesiella, Desulfovibrio, Dorea and Turicibacter* are all included in the collection of HMP microbial reference genomes.

The two most prevalent novel taxa we detected are in the genus *Barnesiella*. OTU 12 was found in a single subject and OTU 13 was found in eight subjects. These two OTUs were found only in samples that were collected in Houston, suggesting they may be suited to bacterial communities related to specific environmental or dietary factors. *Barnesiella* was recently discovered in samples from the chicken gut [18] and in human stool samples [19]. Additional studies are needed to determine the roles of *Barnesiella* species in healthy communities and communities affected by diet or disease.

Some of the other novel taxa we detected in these stool samples are related to genera associated with shifts in the microbiome related to diet (*Oscillibacter*) [20], colorectal adenoma (*Dorea*) [21], and opportunistic infections (*Desulfovibrio*) [22]. More thorough characterization and whole-genome sequencing of the novel taxa we identified will be important in order to determine if each of the novel taxa constitute new families, genera or species and if they affect or fluctuate with human health conditions.

A better understanding of the effects of bacteria that are less abundant, less broadly distributed and less well characterized is important as we seek to understand the complex gut microbiome in healthy individuals and link changes in the microbiome to disease. Furthermore, the pipelines and analysis criteria we developed can be applied to the analysis of novel 16S rDNA sequences in other microbial communities or habitats, which may be less well characterized than stool. The HMP has generated sequence data from samples taken from oral, skin, nasal, and vaginal body sites in addition to stool, and we anticipate that the examination of the bacterial communities in those body sites may reveal diversity that has yet to be appreciated.

## Methods

### Samples

As part of the HMP initiative [8], subjects provided stool samples at one, two or three visits, spaced 30 to 359 days apart (Aargard and Petrosino et al, manuscript submitted). DNA extracted from each sample (Aargard and Petrosino et al, manuscript submitted) was sequenced to generate the datasets described below (The Human Microbiome Consortium (2012) ‘Structure, Function and Diversity of Human Microbiome in an Adult Reference Population’. Nature: doi:10.1038/nature11234). All datasets are available from the Short Read Archive (http://www.ncbi.nlm.nih.gov/sra). Accession numbers are given for WGS samples in the supplemental materials. Variable region samples were pooled before sequencing, and the datasets can be downloaded with the study name “Human Microbiome Project 16S rRNA 454 Clinical Production Phase I.”

### Whole-genome Shotgun Sequences Sequenced on the Roche 454 FLX Titanium Platform (Roche WGS 16S)

All available Roche WGS data generated by the Human Microbiome Consortium were downloaded from the Short Read Archive (http://www.ncbi.nlm.nih.gov/sra). Data had previously been processed to remove human sequences. Analysis was limited to stool samples for which there also existed Roche variable region data. This yielded 12 samples from 11 individuals; short read archive identifiers are listed in Table S1. From this dataset, the run SRR063897 from male subject 686765762 was excluded due to a potential body site mislabeling. The RDPclassifier identifies 65% of the reads in this run as the genera *Mobiluncus* or *Atopobium*, taxa typically associated with the oral and vaginal microbiomes.

The remaining 33,236,142 reads were processed with a streamlined version of PhylOTU ([12], improvements discussed below) to identify the 16S sequences and to assess the overall distribution of the Roche WGS reads into OTUs. PhylOTU identifies 16S metagenomic reads through a sequence similarity search against a phylogenetically diverse 16S database (STAP). Bases from the ends of each read that are not significantly similar to a database sequence are trimmed from the read to minimize the inclusion of non-homologous 16S sequence in the subsequent phylogenetic analysis. Of the input reads, 52,756 ranging from 100 to about 500 bases in length were found to align to the 16S locus and were processed further. PhylOTU identified 1361 OTUs from the 16S rDNA sequences, as shown in Figure S2. These 52,756 16S rDNA sequences compose the “Roche WGS” dataset that was used for the discovery of novel sequences.

### Streamlining of PhylOTU

To analyze the Roche WGS dataset, which is approximately three times the size of the dataset on which PhylOTU was benchmarked, PhylOTU was modified to more efficiently process large datasets. Several steps in the PhylOTU workflow were parallelized and can run on a user specified number of nodes on a computing cluster. These include the 16S sequence similarity search, the multiple sequence alignment quality control, and the construction of a phylogenitic distance matrix. An option was implemented to use the hcluster function from the ESPRIT package [23] for the clustering, since it quickly handles clustering a large number of sequences. These combined improvements allowed the Roche WGS dataset to be run in approximately 24 hours. This updated version of PhylOTU is available at https://github.com/sharpton/PhylOTU.

### Whole-genome Shotgun Sequences Generated on the Illumina GAIIX Platform (Illumina WGS 16S)

The Human Microbiome Consortium generated 60–100-base, paired-end Illumina GAIIX data for 147 stool samples. Data downloaded from the Short Read Archive had been processed to trim low-quality sequences, remove duplicate sequences from each sample, mask low-complexity sequences with Dust [24], and remove human sequences (The Human Microbiome Consortium (2012) ‘Structure, Function and Diversity of Human Microbiome in an Adult Reference Population’. Nature: doi:10.1038/nature11234). Short-read archive identifiers for the 147 samples are included in Table S2. Sequences corresponding to bacterial 16S genes were identified by aligning the Illumina WGS sequences to a database of 16S sequences. Alignments were done using cross_match [25] with relaxed parameters: -raw -tags -bandwidth 3 -penalty -1 -gap_init -1 -gap_ext -1 -masklevel 101. The database comprised 12,627 sequences generated by combining the 16S reads from the RDP training set 6 with additional 16S sequences from completed bacterial genomes found at the Entrez Genome Project (http://www.ncbi.nlm.nih.gov/sites/entrez?db=genome) as of August 3, 2010. The 67,679,702 16S sequences were used for further analysis.

### V1–3 and V3–5 16S Variable Region Sequences (Roche V1–3 and Roche V3–5)

Variable region sequences that were generated on the Roche 454 FLX Titanium platform by the HMP Consortium were downloaded from the DACC (Version 1.0) (http://www.hmpdacc.org/resources/data_browser.php) and supplemented with data from (http://www.hmpdacc.org/resources/data_browser.php). The V1–3 dataset included 92,330 reads from 178 stool samples from 139 individuals. The V3–5 dataset included 160,948 reads from 307 stool samples from 208 individuals. Sequences from the V1–3 and V3–5 datasets ranged in length from 200 to 500 bases. The data were minimally processed to remove PCR primer sequences and tags. Chimera screening was done using Chimera Slayer [11], and sequences identified as chimeric were removed from further analysis.

### Identification of Potentially Novel Sequences

Samples from 11 subjects were sequenced by three methods: Roche WGS, Roche targeted 16S variable region, and Illumina WGS. Novel sequences were identified in this subset of samples, which included 12 Roche V1–3 samples from eight of the 11 subjects, 20 Roche V3–5 samples from 11 of the 11 subjects, and 12 Roche WGS samples from 11 of the 11 subjects (Table S2) by the following steps (Figure 1).

Sequences in the Roche V1–3 and V3–5 datasets were clustered to collapse sequences with 100% identity over 95% of the read length using cd-hit [26] and a single representative sequence from each cluster was used in the following. Reads from all three datasets were aligned to (a) the RDP database (downloaded November 9, 2010 from https://rdp.cme.msu.edu, 1356230 sequences) and (b) the NT nucleotide database (last validated with download from June 2011). WU-BLAST was used with default parameters [27].

For the variable region data reads with greater than 97% identity to a sequence in the RDP or NT database over at least 95% of the sequence length were excluded from further analysis, and the remaining sequences were novel candidates. At the end of the analysis pipeline, the nearest neighbor of each novel sequence with Illumina confirmation was identified as the database sequence that aligned to the variable region sequence with the highest bitscore, after requiring the alignment length be at least 100 bp in length and any unaligned portion of the read be ≤5 bp or overhanging the end of the target read.

In the Roche WGS dataset, alignments between a query read and database target were required to be at least 100 bp in length, with any unaligned portion of the read being ≤5 bp in length or overhanging the end of the target read. Of the target alignments meeting these criteria, the one with the highest bitscore was chosen as the nearest neighbor. The Roche WGS reads having a nearest neighbor with <97% identity were then aligned to the variable region primers with NCBI BLAST, using a word size of 7. We reevaluated those sequences with less than 100 bases of consecutive non-primer sequence for a nearest neighbor by relaxing the alignment length from 100 bases to the longest stretch of non-primer sequence found in the read. This check only affects short reads of approximately 100 bases by ensuring that database sequences, which are highly similar to the query except for the primer sequence (which is often trimmed off sequences deposited in the databases), would be considered in the nearest-neighbor search. This also ensures that short reads have high information content and are not identical to a known sequence over a large fraction of the read length. Roche variable region and WGS reads having a nearest neighbor with <97% identity were considered potentially novel in the initial analysis.

### Validating Novel 16S Sequences from Roche WGS and Roche V1–3 and V3–5 Datasets with Illumina WGS Reads

Potentially novel sequences were confirmed by aligning the Illumina WGS sequences from all of the stool samples to the potentially novel sequences from the Roche variable region datasets or Roche WGS data with cross_match [25]. For the Roche WGS read, alignments were required to be 100% identical and at least 60 bp in length. The entire length of the Illumina read was required to align, excluding any portion extending beyond the end of the Roche WGS read. Only Roche WGS reads that were covered by aligned Illumina reads over 100% of their length were retained for further analysis. For the Roche variable region data, Illumina WGS sequences that aligned with 100% identity over 100% of their length were used to validate the sequences. The depth and breadth of coverage of the Roche variable region read with Illumina WGS reads was evaluated using RefCov [28]. Roche variable region reads that were covered by aligned Illumina WGS reads over greater than 95% of their sequence length were included for further analysis. The 95% cutoff was used for the Roche variable region sequences because the sequences had not been subject to stringent quality trimming and, therefore, might contain some sequencing error on the ends of the reads.

Illumina sequence coverage of Roche WGS and variable region sequences was evaluated as follows. We observed two major irregularities that were indicative of chimerism, miscalled bases, uncalled bases, insertions, deletions, or insufficient Illumina coverage. First, for a Roche read with good Illumina support, the maximum length of the Illumina WGS sequences (60–100 bases in length) that were tiled across the Roche sequence remained a fairly constant length (>90 bases) at any given alignment start point (except at the end of the read). In contrast, the maximum read lengths of the tiled Illumina reads covering a problematic read gradually decreased until the maximum read length was ≤70 bases, only to increase again (Figure 2). Second, for a Roche read with good Illumina support, a tiling path could be identified consisting of Illumina sequences that overlapped by at least 20 bases and extended the coverage by at least 20 bases (excluding those that aligned at the end of the Roche sequence). However, this was not true for Roche reads that were problematic or lacked sufficient Illumina support (Figure 2). The chimeric sequence analysis included manual evaluation of hundreds of reads, including the alignment of separate pieces of the reads to different taxa in 16S rDNA sequence databases. Alignments and coverage were manually reviewed using Tablet [29].

Potentially novel reads were further evaluated for a third irregularity in the Illumina coverage using the paired-end feature (sequences from both ends of the same DNA fragment) of the Illumina WGS data (Figure 2). Some of the paired sequences should (1) align to a non-chimeric Roche variable region sequence or high-quality Roche WGS sequence and (2) span nearly the full length of the Roche read. Roche sequences that do not have support from paired-end Illumina reads could be chimeric or have sequencing errors that prohibit the alignment of one of the mate pairs. Therefore, the paired-end test provides additional support for a Roche sequence that shows no other evidence of chimerism or error. This paired-end test is not informative for all Roche reads, as only a subset of paired end sequences was expected to span the length of the Roche variable region and WGS sequences for several reasons. First, the insert sizes of Illumina WGS libraries ranged from 100–500 bases, so only those paired reads from a fragment of approximately the length of the Roche read being examined could provide support. Furthermore, Roche sequences with few supporting Illumina WGS reads, which are likely rare sequences, may not have paired-end sequences due to the decreased likelihood of finding paired ends with the right span within the relatively few aligned Illumina sequences. Therefore, we only required paired-end support for those reads with a median depth of Illumina WGS coverage greater than 500 reads and a length of greater than 200 bp. These reads were only considered high-quality novel sequences if a set of Illumina WGS paired end reads spanned at least 90% of the Roche sequence.

### Identification of Nearest Neighbors to Novel Reads from Cultured Bacteria

A nearest neighbor was identified for each novel read from subset of reads in the databases that were labeled to indicate they were from a cultured source. Nearest neighbors from these smaller databases were identified in the same way as above. Alignments were required to be at least 100 bp in length and any unaligned portion of the sequence to be ≤5 bp or overhanging the end of the target read. The nearest neighbor was chosen as the highest bitscore alignment meeting these criteria.

For the RDP database, all reads marked as Source:Isolates and Quality:Good were considered. For the NT database, reads were excluded based on the sequence description. All sequences with “uncultured” or “16S rDNA sequence amplified from human fecal sample” in the description were excluded. The descriptions of nearest-neighbor hits were then manually inspected and those sequences from uncultured sources were excluded.

### Taxonomic Classification of Novel Reads

Novel reads were classified by the RDP Classifier (version 2.2 using training set 6) [30], which assigns reads to taxonomic groups from the phylum to genus level and generates a bootstrap value, indicating the confidence of the identification at each taxonomic level. Where applicable, a bootstrap threshold of 0.5 was used.

### Insertion of Novel Sequences into the SILVA Reference Tree and Tree Visualization

Novel sequences were aligned to the full-length SILVA reference database with Mothur [31]. The alignments containing the full-length SILVA reference sequences and novel sequences were transformed to relaxed PHYLIP format. The novel sequences were assigned to the SILVA reference tree by EPA [32] using the maximum-likelihood (ML) method implemented in the RAxML web-based server [33]. Taxonomy tags indicating the phylum of each node were added to the tree with a custom Perl script. The resulting phylogeny was visualized and edited with Archaeopteryx (version 0.957 beta, http://www.phylosoft.org/archaeopteryx) [34].

### Classification of Novel Reads into OTUs

Novel sequences from the Roche variable region and WGS datasets were clustered using PhylOTU. To eliminate any trimming of the Roche variable region sequences, the first blast step of PhylOTU was skipped with the “–k A” option. The 141 novel Roche WGS and variable region sequences were clustered into 26 operational taxonomic units (OTUs) with PhylOTU (Table S3). The two largest OTUs (containing 12 and 89 sequences, respectively) contained sequences from both the Roche WGS and at least one Roche variable region dataset. The remaining OTUs contained less than six sequences.

### Analysis of Primer Sequences in the Roche WGS Novel Sequences

To address the effects of primer bias on the datasets, we determined if the reads detected in the Roche WGS dataset could have been detected with the primers used to produce the variable region datasets. Aligning the Roche WGS novel sequences (with NCBI BLAST, using a word size of 7) to the primers used in the V1–3, V3–5 and V6–9 variable-region sequencing identified 78 of the 85 reads with 100% identity to one of the primer sequences. Three reads did not overlap any of the variable region primer sites, but the sequences aligned to sequences within the amplified variable region datasets, indicating that the novel sequences are within a region that can be amplified. The remaining four reads had one or two mismatches to a primer, but would likely still amplify. The Roche WGS dataset did not reveal any obvious primer bias. However, the identification of 16S rDNA sequences from the Roche WGS data and the associated trimming may bias the Roche WGS data sample toward known sequences, so that novel reads with unique sequence at the priming sites were excluded from this analysis early in the analysis pipeline.

### Distribution of Novel Sequences in an Extended Dataset

In the Roche variable region data, the longest read from each OTU was chosen as a representative sequence for the OTU. The representative sequences were clustered with data from 131 and 197 additional subjects (Table S4) for the V1–3 and V3–5 data, respectively, using cd-hit [26] at a 97% identity threshold. Sequences co-clustering with the representative sequences were used as an approximation of the presence of the representative novel sequence in additional subjects. Roche WGS data were not available from a substantial number of additional subjects, so we used a different approach based on Illumina WGS data (147 samples from the 94 subjects represented in the variable region datasets). If an Illumina WGS sample contained reads validating 100% of a Roche WGS sequence from a novel OTU, we concluded that the novel OTU was present in the Illumina sample. These methods yielded a conservative estimate of the distribution of the novel taxa among individuals.

## Supporting Information

Figure S1 **Graphical descriptions of datasets.**
(TIF)

Figure S2 **Distribution of Roche WGS OTUs across individuals.** All Roche WGS reads identified as 16S rDNA were clustered into 1361 species-level OTUs with PhylOTU. The fraction of OTUs found in each subject is shown, indicating the relative diversity of the samples from each subject. “V1” and “V2” indicate two independent visits at which the subject 809635352 was sampled.
(TIF)

Table S1 **Roche samples evaluated for novel taxa.**
(DOC)

Table S2 **Short-read archive IDs for the Illumina WGS dataset.**
(DOC)

Table S3 **Table describing read attributes, nearest neighbors, RDP calls.**
(XLSX)

Table S4 **Subject and study sample IDs for the expanded variable region datasets.**
(DOC)

Text S1 **Comparison of novel sequences from Roche WGS and variable region datasets.**
(DOC)

## References

[1] Dethlefsen L, Huse S, Sogin ML, Relman DA (2008). The pervasive effects of an antibiotic on the human gut microbiota, as revealed by deep 16S rRNA sequencing.. PLoS Biol.

[2] Arumugam M, Raes J, Pelletier E, Le Paslier D, Yamada T (2011). Enterotypes of the human gut microbiome.. Nature.

[3] Suau A, Bonnet R, Sutren M, Godon JJ, Gibson GR (1999). Direct analysis of genes encoding 16S rRNA from complex communities reveals many novel molecular species within the human gut.. Appl Environ Microbiol.

[4] Eckburg PB, Bik EM, Bernstein CN, Purdom E, Dethlefsen L (2005). Diversity of the human intestinal microbial flora.. Science.

[5] Gill SR, Pop M, Deboy RT, Eckburg PB, Turnbaugh PJ (2006). Metagenomic analysis of the human distal gut microbiome.. Science.

[6] Turnbaugh PJ, Hamady M, Yatsunenko T, Cantarel BL, Duncan A (2009). A core gut microbiome in obese and lean twins.. Nature.

[7] Kurokawa K, Itoh T, Kuwahara T, Oshima K, Toh H (2007). Comparative metagenomics revealed commonly enriched gene sets in human gut microbiomes.. DNA Res.

[8] Peterson J, Garges S, Giovanni M, McInnes P, Wang L (2009). The NIH Human Microbiome Project.. Genome Res.

[9] Cole JR, Chai B, Farris RJ, Wang Q, Kulam-Syed-Mohideen AS (2007). The ribosomal database project (RDP-II): introducing myRDP space and quality controlled public data.. Nucleic Acids Res.

[10] Cole JR, Wang Q, Cardenas E, Fish J, Chai B (2009). The Ribosomal Database Project: improved alignments and new tools for rRNA analysis.. Nucleic Acids Res.

[11] Haas BJ, Gevers D, Earl AM, Feldgarden M, Ward DV (2011). Chimeric 16S rRNA sequence formation and detection in Sanger and 454-pyrosequenced PCR amplicons.. Genome Res.

[12] Sharpton TJ, Riesenfeld SJ, Kembel SW, Ladau J, O’Dwyer JP (2011). PhylOTU: a high-throughput procedure quantifies microbial community diversity and resolves novel taxa from metagenomic data.. PloS Comput Biol.

[13] Altschul SF, Madden TL, Schaffer AA, Zhang J, Zhang Z (1997). Gapped BLAST and PSI-BLAST: a new generation of protein database search programs.. Nucleic Acids Res.

[14] Hugenholtzt P, Huber T (2003). Chimeric 16S rDNA sequences of diverse origin are accumulating in the public databases.. Int J Syst Evol Microbiol.

[15] Costello EK, Lauber CL, Hamady M, Fierer N, Gordon JI (2009). Bacterial community variation in human body habitats across space and time.. Science.

[16] Hajishengallis G, Liang S, Payne MA, Hashim A, Jotwani R (2011). Low-Abundance Biofilm Species Orchestrates Inflammatory Periodontal Disease through the Commensal Microbiota and Complement.. Cell Host Microbe.

[17] Aronsson B, Mollby R, Nord CE (1981). Occurrence of toxin-producing Clostridium difficile in antibiotic-associated diarrhea in Sweden.. Med Microbiol Immunol.

[18] Sakamoto M, Lan PT, Benno Y (2007). Barnesiella viscericola gen. nov., sp. nov., a novel member of the family Porphyromonadaceae isolated from chicken caecum.. Int J Syst Evol Microbiol.

[19] Morotomi M, Nagai F, Sakon H, Tanaka R (2008). Dialister succinatiphilus sp. nov. and Barnesiella intestinihominis sp. nov., isolated from human faeces.. Int J Syst Evol Microbiol.

[20] Walker AW, Ince J, Duncan SH, Webster LM, Holtrop G (2011). Dominant and diet-responsive groups of bacteria within the human colonic microbiota.. ISME J.

[21] Shen XJ, Rawls JF, Randall T, Burcal L, Mpande CN (2010). Molecular characterization of mucosal adherent bacteria and associations with colorectal adenomas.. Gut Microbes.

[22] Goldstein EJ, Citron DM, Peraino VA, Cross SA (2003). Desulfovibrio desulfuricans bacteremia and review of human Desulfovibrio infections.. J Clin Microbiol.

[23] Sun Y, Cai Y, Liu L, Yu F, Farrell ML (2009). ESPRIT: estimating species richness using large collections of 16S rRNA pyrosequences.. Nucleic Acids Res.

[24] Morgulis A, Gertz EM, Schaffer AA, Agarwala R (2006). A fast and symmetric DUST implementation to mask low-complexity DNA sequences.. J Comput Biol.

[25] Green P (1994). Cross_match.. http://www.phrap.org.

[26] Li W, Godzik A (2006). Cd-hit: a fast program for clustering and comparing large sets of protein or nucleotide sequences.. Bioinformatics.

[27] Altschul SF, Gish W, Miller W, Myers EW, Lipman DJ (1990). Basic local alignment search tool.. J Mol Biol.

[28] Wylie TN, Walker J, Mardis ER Refcov version 2.0.. http://gmt.genome.wustl.edu/gmt-refcov.

[29] Milne I, Bayer M, Cardle L, Shaw P, Stephen G (2010). Tablet–next generation sequence assembly visualization.. Bioinformatics.

[30] Wang Q, Garrity GM, Tiedje JM, Cole JR (2007). Naive Bayesian classifier for rapid assignment of rRNA sequences into the new bacterial taxonomy.. Appl Environ Microbiol.

[31] Schloss PD, Westcott SL, Ryabin T, Hall JR, Hartmann M (2009). Introducing mothur: open-source, platform-independent, community-supported software for describing and comparing microbial communities.. Appl Environ Microbiol.

[32] Berger SA, Krompass D, Stamatakis A (2011). Performance, accuracy, and Web server for evolutionary placement of short sequence reads under maximum likelihood.. Syst Biol.

[33] Stamatakis A, Ludwig T, Meier H (2005). RAxML-III: a fast program for maximum likelihood-based inference of large phylogenetic trees.. Bioinformatics.

[34] Han MV, Zmasek CM (2009). phyloXML: XML for evolutionary biology and comparative genomics.. BMC Bioinformatics.

