# Supplementary figures and images for: Novel Bacterial Taxa in the Human Microbiome
Source: PLoS One. 2012 Jun 13;7(6):e35294. doi: 10.1371/journal.pone.0035294 (PMC3374617; doi:10.1371/journal.pone.0035294)

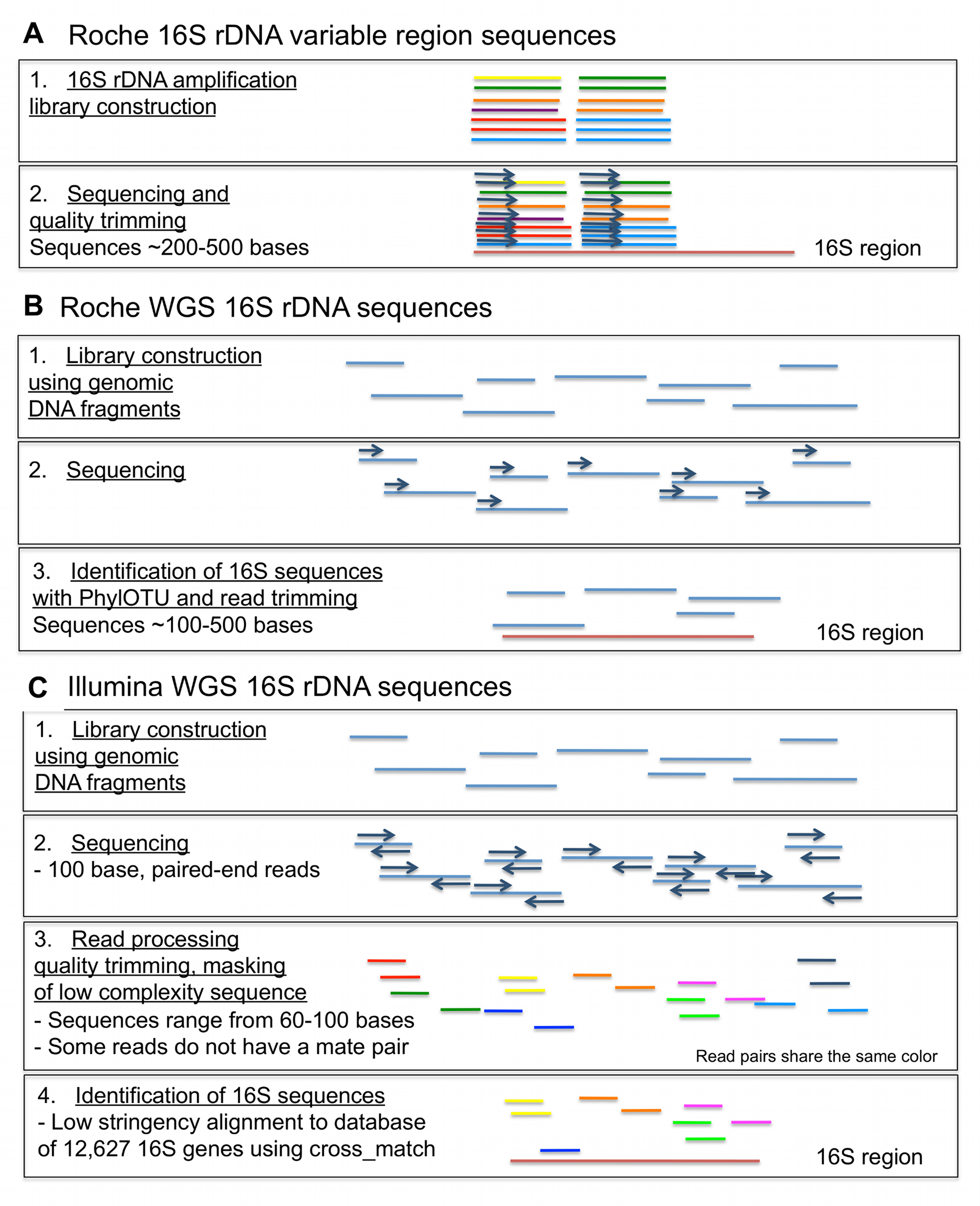

Supplement: Figure S1 — Graphical descriptions of datasets. (TIF) [file pone.0035294.s001.tif]

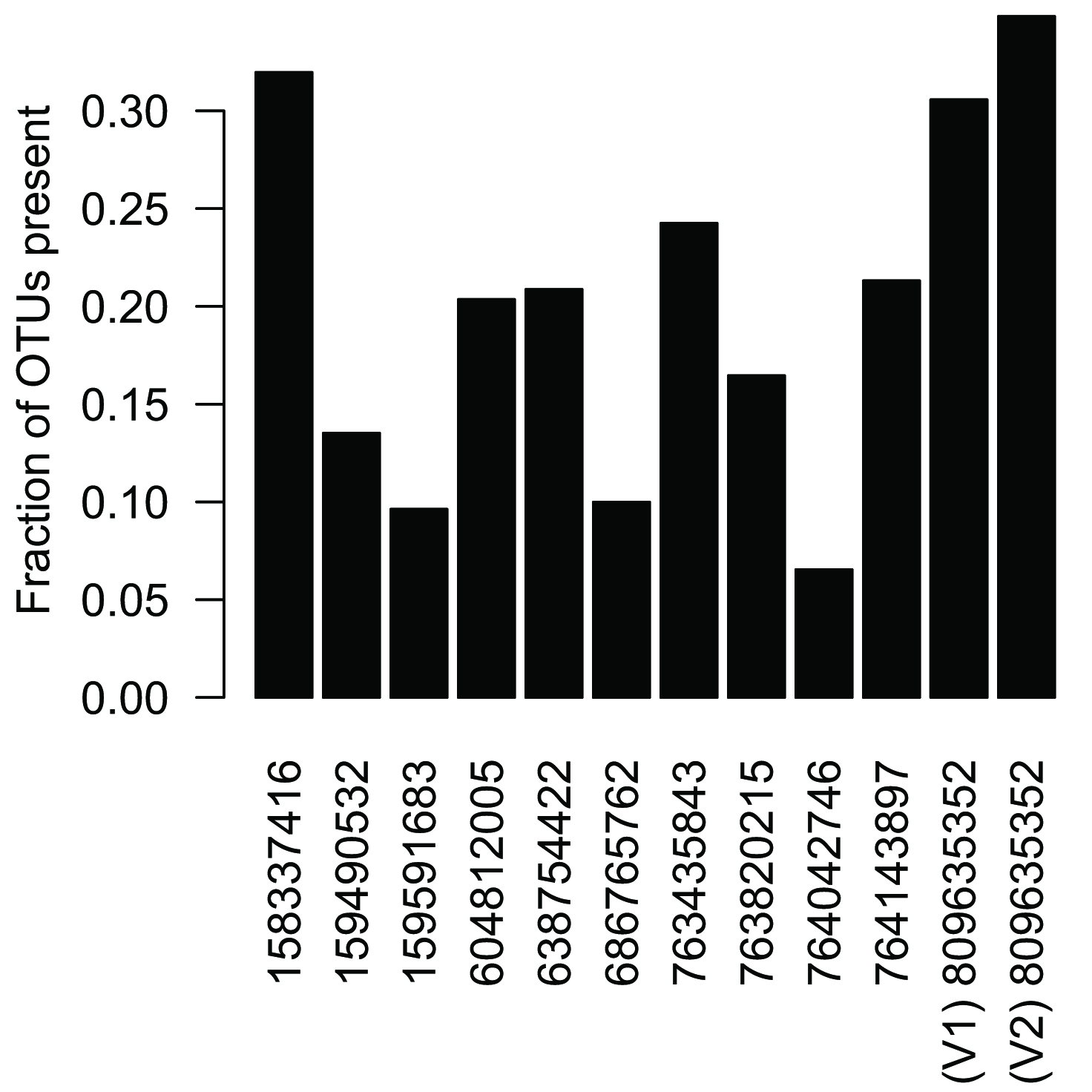

Supplement: Figure S2 — Distribution of Roche WGS OTUs across individuals. All Roche WGS reads identified as 16S rDNA were clustered into 1361 species-level OTUs with PhylOTU. The fraction of OTUs found in each subject is shown, indicating the relative diversity of the samples from each subject. “V1” and “V2” indicate two independent visits at which the subject 809635352 was sampled. (TIF) [file pone.0035294.s002.tif]
