# Supplementary material for: Novel Bacterial Taxa in the Human Microbiome
Source: PLoS One. 2012 Jun 13;7(6):e35294. doi: 10.1371/journal.pone.0035294 (PMC3374617; doi:10.1371/journal.pone.0035294)
Supplement: Table S1 — Roche samples evaluated for novel taxa. (DOC) [file pone.0035294.s003.doc]

**Table S1. Roche samples evaluated for novel taxa.**

| Subject ID | Roche WGS SRA Accession ID | Roche V1-3* Study sample ID | Roche V3-5* Study sample ID |
| --- | --- | --- | --- |
| 158337416 | SRX025494 | Not available | 700097859 |
| 159490532 | SRX025493 | Not available | 700033503, 700102375 |
| 159591683 | SRX025444 | Not available | 700015923, 700101859 |
| 604812005 | SRX025496 | 700110438 | 700102318, 700110438 |
| 638754422 | SRX024348 | 700109252 | 700097928, 700109252 |
| 686765762 | SRX025492 | 700107208, 700111045 | 700107208, 700111045 |
| 763435843 | SRX025207 | 700024470 | 700024470 |
| 763820215 | SRX025209 | 700021934, 700105238 | 700021934, 700105238 |
| 764042746 | SRX025210 | 700024044, 700114480 | 700024044, 700114480 |
| 764143897 | SRX025206 | 700023902, 700106837 | 700023902, 700106837 |
| 809635352 | SRX025495, SRX025497 | 700110834 | 700098451, 700110834 |

* Roche variable region data sets can be downloaded from the Short Read Archive (<http://www.ncbi.nlm.nih.gov/sra>) using the study name, “Human Microbiome Project 16S rRNA 454 Clinical Production Phase I.”
