# Supplementary material for: Novel Bacterial Taxa in the Human Microbiome
Source: PLoS One. 2012 Jun 13;7(6):e35294. doi: 10.1371/journal.pone.0035294 (PMC3374617; doi:10.1371/journal.pone.0035294)
Supplement: Table S2 — Short-read archive IDs for the Illumina WGS dataset. (DOC) [file pone.0035294.s004.doc]

**Table S2. Short-read archive IDs for the Illumina WGS dataset.**

| Subject ID | Short-Read Archive ID |
| --- | --- |
| 158337416 | SRS012273 |
| 158337416 | SRS022071 |
| 158458797 | SRS011061 |
| 158479027 | SRS011084 |
| 158479027 | SRS021484 |
| 158499257 | SRS011134 |
| 158499257 | SRS022609 |
| 158742018 | SRS011239 |
| 158802708 | SRS011271 |
| 158802708 | SRS023526 |
| 158883629 | SRS012902 |
| 158883629 | SRS023829 |
| 158883629 | SRS075398 |
| 158944319 | SRS011302 |
| 158944319 | SRS024265 |
| 159005010 | SRS023583 |
| 159146620 | SRS011452 |
| 159166850 | SRS011529 |
| 159207311 | SRS013476 |
| 159207311 | SRS024132 |
| 159227541 | SRS013521 |
| 159227541 | SRS049995 |
| 159227541 | SRS078176 |
| 159247771 | SRS011405 |
| 159247771 | SRS024009 |
| 159268001 | SRS013687 |
| 159268001 | SRS023914 |
| 159369152 | SRS013158 |
| 159369152 | SRS023971 |
| 159490532 | SRS016989 |
| 159490532 | SRS052027 |
| 159510762 | SRS017247 |
| 159510762 | SRS024075 |
| 159551223 | SRS017103 |
| 159551223 | SRS024331 |
| 159571453 | SRS013800 |
| 159571453 | SRS024435 |
| 159591683 | SRS013215 |
| 159591683 | SRS024549 |
| 159611913 | SRS016954 |
| 159611913 | SRS024625 |
| 159632143 | SRS017307 |
| 159713063 | SRS024388 |
| 159733294 | SRS011586 |
| 159753524 | SRS016495 |
| 159753524 | SRS016517 |
| 159753524 | SRS053214 |
| 159753524 | SRS077730 |
| 159814214 | SRS017191 |
| 159814214 | SRS043701 |
| 159915365 | SRS016585 |
| 160158126 | SRS018133 |
| 160158126 | SRS058770 |
| 160178356 | SRS018351 |
| 160218816 | SRS018427 |
| 160319967 | SRS017433 |
| 160380657 | SRS018313 |
| 160400887 | SRS020233 |
| 160421117 | SRS016753 |
| 160502038 | SRS017521 |
| 160582958 | SRS017701 |
| 160603188 | SRS017821 |
| 160643649 | SRS019968 |
| 160704339 | SRS020328 |
| 160765029 | SRS020869 |
| 246515023 | SRS023346 |
| 246515023 | SRS057717 |
| 338793263 | SRS054590 |
| 370425937 | SRS022713 |
| 370425937 | SRS057478 |
| 404239096 | SRS045713 |
| 508703490 | SRS053335 |
| 550534656 | SRS043001 |
| 604812005 | SRS045004 |
| 604812005 | SRS045645 |
| 638754422 | SRS022137 |
| 638754422 | SRS055982 |
| 675950834 | SRS053398 |
| 686765762 | SRS049900 |
| 686765762 | SRS064645 |
| 706846339 | SRS052697 |
| 737052003 | SRS054956 |
| 763435843 | SRS015794 |
| 763496533 | SRS013951 |
| 763496533 | SRS019161 |
| 763536994 | SRS014287 |
| 763536994 | SRS050422 |
| 763536994 | SRS062427 |
| 763577454 | SRS014459 |
| 763577454 | SRS015065 |
| 763597684 | SRS019582 |
| 763678604 | SRS014235 |
| 763678604 | SRS019685 |
| 763759525 | SRS015190 |
| 763759525 | SRS043411 |
| 763820215 | SRS014313 |
| 763820215 | SRS058723 |
| 763840445 | SRS014613 |
| 763840445 | SRS064276 |
| 763860675 | SRS014923 |
| 763860675 | SRS050925 |
| 763901136 | SRS015133 |
| 763901136 | SRS063985 |
| 763961826 | SRS014683 |
| 763961826 | SRS019030 |
| 763982056 | SRS015264 |
| 763982056 | SRS050299 |
| 764002286 | SRS015854 |
| 764042746 | SRS015369 |
| 764042746 | SRS064557 |
| 764062976 | SRS014979 |
| 764062976 | SRS065504 |
| 764143897 | SRS015217 |
| 764143897 | SRS051882 |
| 764184357 | SRS048870 |
| 764224817 | SRS015782 |
| 764224817 | SRS047044 |
| 764285508 | SRS015578 |
| 764325968 | SRS015960 |
| 764325968 | SRS056259 |
| 764447348 | SRS016018 |
| 764447348 | SRS042628 |
| 764487809 | SRS015663 |
| 764487809 | SRS051031 |
| 764508039 | SRS016056 |
| 764588959 | SRS016095 |
| 764649650 | SRS016203 |
| 764669880 | SRS016267 |
| 764669880 | SRS019787 |
| 764811490 | SRS042284 |
| 764892411 | SRS019910 |
| 765013792 | SRS018656 |
| 765034022 | SRS019267 |
| 765074482 | SRS016335 |
| 765074482 | SRS047014 |
| 765074482 | SRS049164 |
| 765094712 | SRS018575 |
| 765094712 | SRS056519 |
| 765135172 | SRS018817 |
| 765560005 | SRS019397 |
| 765620695 | SRS019601 |
| 765640925 | SRS050752 |
| 765701615 | SRS049959 |
| 809635352 | SRS022524 |
| 809635352 | SRS063040 |
| 823052294 | SRS049712 |
| 861967750 | SRS048164 |
