# Supplementary material for: Novel Bacterial Taxa in the Human Microbiome
Source: PLoS One. 2012 Jun 13;7(6):e35294. doi: 10.1371/journal.pone.0035294 (PMC3374617; doi:10.1371/journal.pone.0035294)
Supplement: Table S4 — Subject and study sample IDs for the expanded variable region datasets. (DOC) [file pone.0035294.s006.doc]

**Table S4. Subject and study sample IDs for the expanded variable region datasets.**

| Variable region | Subject ID | Study sample ID |
| --- | --- | --- |
| V1-3 | 132902142 | 700103499 |
| V1-3 | 147406386 | 700106978 |
| V1-3 | 158013734 | 700013596 |
| V1-3 | 158013734 | 700097313 |
| V1-3 | 158114885 | 700099021 |
| V1-3 | 158155345 | 700097650 |
| V1-3 | 158216035 | 700097253 |
| V1-3 | 158236265 | 700098954 |
| V1-3 | 158276726 | 700099081 |
| V1-3 | 158398106 | 700095372 |
| V1-3 | 158418336 | 700095429 |
| V1-3 | 158438567 | 700099609 |
| V1-3 | 158458797 | 700098381 |
| V1-3 | 158802708 | 700015250 |
| V1-3 | 158822939 | 700014956 |
| V1-3 | 158883629 | 700100249 |
| V1-3 | 158924089 | 700015407 |
| V1-3 | 158964549 | 700016012 |
| V1-3 | 158964549 | 700099765 |
| V1-3 | 159005010 | 700016021 |
| V1-3 | 159085930 | 700016029 |
| V1-3 | 159146620 | 700016142 |
| V1-3 | 159146620 | 700102924 |
| V1-3 | 159166850 | 700016610 |
| V1-3 | 159166850 | 700102261 |
| V1-3 | 159227541 | 700016564 |
| V1-3 | 159227541 | 700102607 |
| V1-3 | 159247771 | 700015981 |
| V1-3 | 159389382 | 700016921 |
| V1-3 | 159389382 | 700101657 |
| V1-3 | 159470302 | 700033438 |
| V1-3 | 159713063 | 700033749 |
| V1-3 | 159733294 | 700033153 |
| V1-3 | 160744799 | 700096656 |
| V1-3 | 160825720 | 700095793 |
| V1-3 | 160825720 | 700108304 |
| V1-3 | 160866180 | 700096612 |
| V1-3 | 160866180 | 700109340 |
| V1-3 | 160886410 | 700096285 |
| V1-3 | 160906640 | 700095975 |
| V1-3 | 160947100 | 700096066 |
| V1-3 | 160947100 | 700109827 |
| V1-3 | 160967330 | 700097135 |
| V1-3 | 160967330 | 700108493 |
| V1-3 | 161007791 | 700108977 |
| V1-3 | 161270782 | 700097589 |
| V1-3 | 161270782 | 700108058 |
| V1-3 | 161311242 | 700107836 |
| V1-3 | 161331472 | 700109770 |
| V1-3 | 161473083 | 700110243 |
| V1-3 | 178713055 | 700105087 |
| V1-3 | 184349034 | 700106723 |
| V1-3 | 208027353 | 700106579 |
| V1-3 | 246515023 | 700110177 |
| V1-3 | 275382046 | 700105001 |
| V1-3 | 289996019 | 700106350 |
| V1-3 | 289996019 | 700114179 |
| V1-3 | 295137534 | 700110606 |
| V1-3 | 336497421 | 700111524 |
| V1-3 | 338793263 | 700106465 |
| V1-3 | 368533040 | 700110663 |
| V1-3 | 370027359 | 700038978 |
| V1-3 | 370425937 | 700109405 |
| V1-3 | 375450439 | 700105822 |
| V1-3 | 375450439 | 700114818 |
| V1-3 | 404239096 | 700038626 |
| V1-3 | 414519462 | 700111833 |
| V1-3 | 465578759 | 700103328 |
| V1-3 | 492786515 | 700110720 |
| V1-3 | 508703490 | 700038761 |
| V1-3 | 516889361 | 700105334 |
| V1-3 | 517810313 | 700106930 |
| V1-3 | 550534656 | 700106326 |
| V1-3 | 553359145 | 700106542 |
| V1-3 | 561079058 | 700114939 |
| V1-3 | 604812005 | 700110438 |
| V1-3 | 612472597 | 700105181 |
| V1-3 | 638754422 | 700109252 |
| V1-3 | 643185023 | 700105496 |
| V1-3 | 650853796 | 700110375 |
| V1-3 | 663835652 | 700114246 |
| V1-3 | 668248235 | 700105904 |
| V1-3 | 675950834 | 700106259 |
| V1-3 | 682102541 | 700114872 |
| V1-3 | 682449369 | 700103327 |
| V1-3 | 686765762 | 700107208 |
| V1-3 | 686765762 | 700111045 |
| V1-3 | 706846339 | 700106170 |
| V1-3 | 737052003 | 700106093 |
| V1-3 | 739574095 | 700114517 |
| V1-3 | 763395383 | 700114017 |
| V1-3 | 763435843 | 700024470 |
| V1-3 | 763456073 | 700105723 |
| V1-3 | 763476303 | 700038520 |
| V1-3 | 763516763 | 700023023 |
| V1-3 | 763516763 | 700105856 |
| V1-3 | 763536994 | 700021876 |
| V1-3 | 763536994 | 700038472 |
| V1-3 | 763536994 | 700113616 |
| V1-3 | 763557224 | 700105459 |
| V1-3 | 763597684 | 700023313 |
| V1-3 | 763597684 | 700038053 |
| V1-3 | 763638144 | 700023226 |
| V1-3 | 763638144 | 700114287 |
| V1-3 | 763698834 | 700023096 |
| V1-3 | 763698834 | 700038740 |
| V1-3 | 763719065 | 700113541 |
| V1-3 | 763759525 | 700023845 |
| V1-3 | 763759525 | 700103710 |
| V1-3 | 763820215 | 700021934 |
| V1-3 | 763820215 | 700105238 |
| V1-3 | 763840445 | 700023267 |
| V1-3 | 763840445 | 700114125 |
| V1-3 | 763860675 | 700023578 |
| V1-3 | 763860675 | 700103478 |
| V1-3 | 763901136 | 700023788 |
| V1-3 | 763901136 | 700114653 |
| V1-3 | 763921366 | 700023477 |
| V1-3 | 763982056 | 700023936 |
| V1-3 | 763982056 | 700106647 |
| V1-3 | 764002286 | 700023539 |
| V1-3 | 764002286 | 700024509 |
| V1-3 | 764042746 | 700024044 |
| V1-3 | 764042746 | 700114480 |
| V1-3 | 764062976 | 700023654 |
| V1-3 | 764083206 | 700024107 |
| V1-3 | 764083206 | 700037560 |
| V1-3 | 764143897 | 700023902 |
| V1-3 | 764143897 | 700106837 |
| V1-3 | 764184357 | 700023987 |
| V1-3 | 764184357 | 700106056 |
| V1-3 | 764224817 | 700024437 |
| V1-3 | 764224817 | 700106695 |
| V1-3 | 764245047 | 700024173 |
| V1-3 | 764285508 | 700024233 |
| V1-3 | 764305738 | 700024566 |
| V1-3 | 764305738 | 700037484 |
| V1-3 | 764325968 | 700024615 |
| V1-3 | 764325968 | 700105306 |
| V1-3 | 764346198 | 700024254 |
| V1-3 | 764346198 | 700113562 |
| V1-3 | 764447348 | 700024673 |
| V1-3 | 764447348 | 700105771 |
| V1-3 | 764467579 | 700024379 |
| V1-3 | 764487809 | 700024318 |
| V1-3 | 764487809 | 700105372 |
| V1-3 | 764508039 | 700024711 |
| V1-3 | 764588959 | 700024752 |
| V1-3 | 764649650 | 700024866 |
| V1-3 | 764669880 | 700024930 |
| V1-3 | 764669880 | 700038263 |
| V1-3 | 764710340 | 700037026 |
| V1-3 | 764750800 | 700113035 |
| V1-3 | 764811490 | 700106914 |
| V1-3 | 764831721 | 700114749 |
| V1-3 | 764872181 | 700113093 |
| V1-3 | 764892411 | 700038386 |
| V1-3 | 764953101 | 700114419 |
| V1-3 | 765013792 | 700037200 |
| V1-3 | 765034022 | 700037738 |
| V1-3 | 765074482 | 700024998 |
| V1-3 | 765074482 | 700105612 |
| V1-3 | 765094712 | 700103653 |
| V1-3 | 765155402 | 700037122 |
| V1-3 | 765195863 | 700037179 |
| V1-3 | 765216093 | 700037389 |
| V1-3 | 765256553 | 700037341 |
| V1-3 | 765276783 | 700037437 |
| V1-3 | 765317243 | 700038005 |
| V1-3 | 765337473 | 700037852 |
| V1-3 | 809635352 | 700110834 |
| V1-3 | 857980665 | 700106784 |
| V1-3 | 863126187 | 700103575 |
| V1-3 | 892969023 | 700110309 |
| V1-3 | 901775393 | 700114767 |
| V1-3 | 937495960 | 700111596 |
| V1-3 | 953045535 | 700105632 |
| V1-3 | 970836795 | 700109129 |
| V3-5 | 147406386 | 700106978 |
| V3-5 | 158013734 | 700013596 |
| V3-5 | 158013734 | 700097313 |
| V3-5 | 158114885 | 700099021 |
| V3-5 | 158216035 | 700097253 |
| V3-5 | 158236265 | 700098954 |
| V3-5 | 158256496 | 700097710 |
| V3-5 | 158276726 | 700099081 |
| V3-5 | 158337416 | 700097859 |
| V3-5 | 158357646 | 700035256 |
| V3-5 | 158398106 | 700095372 |
| V3-5 | 158418336 | 700095429 |
| V3-5 | 158438567 | 700099609 |
| V3-5 | 158458797 | 700098381 |
| V3-5 | 158479027 | 700097196 |
| V3-5 | 158499257 | 700098561 |
| V3-5 | 158721788 | 700100041 |
| V3-5 | 158742018 | 700098289 |
| V3-5 | 158802708 | 700015250 |
| V3-5 | 158802708 | 700099822 |
| V3-5 | 158822939 | 700014956 |
| V3-5 | 158883629 | 700100249 |
| V3-5 | 158924089 | 700015407 |
| V3-5 | 158924089 | 700099307 |
| V3-5 | 158944319 | 700101243 |
| V3-5 | 158964549 | 700016012 |
| V3-5 | 158964549 | 700099765 |
| V3-5 | 159005010 | 700016021 |
| V3-5 | 159005010 | 700099908 |
| V3-5 | 159085930 | 700016029 |
| V3-5 | 159085930 | 700109943 |
| V3-5 | 159146620 | 700016142 |
| V3-5 | 159146620 | 700102924 |
| V3-5 | 159166850 | 700016610 |
| V3-5 | 159166850 | 700102261 |
| V3-5 | 159207311 | 700016470 |
| V3-5 | 159207311 | 700100619 |
| V3-5 | 159227541 | 700016564 |
| V3-5 | 159227541 | 700102607 |
| V3-5 | 159247771 | 700015981 |
| V3-5 | 159247771 | 700100490 |
| V3-5 | 159268001 | 700016765 |
| V3-5 | 159268001 | 700100312 |
| V3-5 | 159288231 | 700016716 |
| V3-5 | 159288231 | 700102867 |
| V3-5 | 159308461 | 700033202 |
| V3-5 | 159308461 | 700101095 |
| V3-5 | 159328691 | 700016214 |
| V3-5 | 159369152 | 700015876 |
| V3-5 | 159389382 | 700016921 |
| V3-5 | 159389382 | 700101657 |
| V3-5 | 159429842 | 700032133 |
| V3-5 | 159450072 | 700033382 |
| V3-5 | 159470302 | 700033438 |
| V3-5 | 159490532 | 700033503 |
| V3-5 | 159490532 | 700102375 |
| V3-5 | 159510762 | 700033941 |
| V3-5 | 159510762 | 700100559 |
| V3-5 | 159510762 | 700112004 |
| V3-5 | 159551223 | 700033665 |
| V3-5 | 159551223 | 700101366 |
| V3-5 | 159571453 | 700016961 |
| V3-5 | 159571453 | 700101600 |
| V3-5 | 159591683 | 700015923 |
| V3-5 | 159591683 | 700101859 |
| V3-5 | 159611913 | 700033435 |
| V3-5 | 159611913 | 700101916 |
| V3-5 | 159632143 | 700033989 |
| V3-5 | 159632143 | 700107508 |
| V3-5 | 159672603 | 700032087 |
| V3-5 | 159672603 | 700101309 |
| V3-5 | 159713063 | 700033749 |
| V3-5 | 159713063 | 700101534 |
| V3-5 | 159733294 | 700033153 |
| V3-5 | 159753524 | 700032244 |
| V3-5 | 159753524 | 700107397 |
| V3-5 | 159814214 | 700033797 |
| V3-5 | 159814214 | 700107059 |
| V3-5 | 159915365 | 700032338 |
| V3-5 | 159915365 | 700108618 |
| V3-5 | 160016515 | 700035392 |
| V3-5 | 160036745 | 700032414 |
| V3-5 | 160056975 | 700035400 |
| V3-5 | 160056975 | 700107949 |
| V3-5 | 160097436 | 700035594 |
| V3-5 | 160097436 | 700107565 |
| V3-5 | 160158126 | 700035533 |
| V3-5 | 160158126 | 700102432 |
| V3-5 | 160178356 | 700035804 |
| V3-5 | 160178356 | 700109582 |
| V3-5 | 160218816 | 700035861 |
| V3-5 | 160218816 | 700108915 |
| V3-5 | 160239046 | 700035321 |
| V3-5 | 160259276 | 700035953 |
| V3-5 | 160319967 | 700034188 |
| V3-5 | 160319967 | 700109643 |
| V3-5 | 160380657 | 700035747 |
| V3-5 | 160380657 | 700107892 |
| V3-5 | 160400887 | 700095486 |
| V3-5 | 160400887 | 700107778 |
| V3-5 | 160421117 | 700032944 |
| V3-5 | 160421117 | 700108180 |
| V3-5 | 160441347 | 700035176 |
| V3-5 | 160461578 | 700034641 |
| V3-5 | 160481808 | 700034100 |
| V3-5 | 160502038 | 700034254 |
| V3-5 | 160502038 | 700108117 |
| V3-5 | 160542498 | 700034857 |
| V3-5 | 160582958 | 700034794 |
| V3-5 | 160582958 | 700108552 |
| V3-5 | 160603188 | 700034926 |
| V3-5 | 160603188 | 700108858 |
| V3-5 | 160643649 | 700095235 |
| V3-5 | 160643649 | 700110111 |
| V3-5 | 160663879 | 700095543 |
| V3-5 | 160684109 | 700095736 |
| V3-5 | 160704339 | 700095669 |
| V3-5 | 160704339 | 700108240 |
| V3-5 | 160744799 | 700096656 |
| V3-5 | 160765029 | 700096380 |
| V3-5 | 160765029 | 700109192 |
| V3-5 | 160825720 | 700095793 |
| V3-5 | 160825720 | 700108304 |
| V3-5 | 160845950 | 700095850 |
| V3-5 | 160845950 | 700109525 |
| V3-5 | 160866180 | 700096612 |
| V3-5 | 160866180 | 700109340 |
| V3-5 | 160886410 | 700096285 |
| V3-5 | 160906640 | 700095975 |
| V3-5 | 160947100 | 700096066 |
| V3-5 | 160947100 | 700109827 |
| V3-5 | 160967330 | 700097135 |
| V3-5 | 160967330 | 700108493 |
| V3-5 | 160987560 | 700096123 |
| V3-5 | 160987560 | 700108802 |
| V3-5 | 161007791 | 700096722 |
| V3-5 | 161007791 | 700108977 |
| V3-5 | 161028021 | 700097022 |
| V3-5 | 161068481 | 700096437 |
| V3-5 | 161230322 | 700096887 |
| V3-5 | 161270782 | 700097589 |
| V3-5 | 161270782 | 700108058 |
| V3-5 | 161311242 | 700096530 |
| V3-5 | 161311242 | 700107836 |
| V3-5 | 161331472 | 700097376 |
| V3-5 | 161331472 | 700109770 |
| V3-5 | 161351702 | 700096962 |
| V3-5 | 161351702 | 700109884 |
| V3-5 | 161412393 | 700096180 |
| V3-5 | 161412393 | 700110007 |
| V3-5 | 161473083 | 700097503 |
| V3-5 | 161473083 | 700110243 |
| V3-5 | 161493313 | 700097437 |
| V3-5 | 161554003 | 700097773 |
| V3-5 | 161554003 | 700109468 |
| V3-5 | 178713055 | 700105087 |
| V3-5 | 184349034 | 700106723 |
| V3-5 | 206906765 | 700099464 |
| V3-5 | 208027353 | 700106579 |
| V3-5 | 246515023 | 700099531 |
| V3-5 | 246515023 | 700110177 |
| V3-5 | 257905678 | 700111178 |
| V3-5 | 275382046 | 700105001 |
| V3-5 | 289996019 | 700106350 |
| V3-5 | 289996019 | 700114179 |
| V3-5 | 295137534 | 700098754 |
| V3-5 | 295137534 | 700110606 |
| V3-5 | 336497421 | 700100184 |
| V3-5 | 336497421 | 700111524 |
| V3-5 | 338793263 | 700106465 |
| V3-5 | 355657046 | 700038950 |
| V3-5 | 368533040 | 700102062 |
| V3-5 | 368533040 | 700110663 |
| V3-5 | 370027359 | 700038978 |
| V3-5 | 370425937 | 700098691 |
| V3-5 | 370425937 | 700109405 |
| V3-5 | 375450439 | 700105822 |
| V3-5 | 375450439 | 700114818 |
| V3-5 | 404239096 | 700038626 |
| V3-5 | 414519462 | 700102135 |
| V3-5 | 414519462 | 700111833 |
| V3-5 | 432193348 | 700098621 |
| V3-5 | 441369442 | 700099237 |
| V3-5 | 451588811 | 700111461 |
| V3-5 | 465578759 | 700103328 |
| V3-5 | 492786515 | 700098220 |
| V3-5 | 492786515 | 700110720 |
| V3-5 | 508703490 | 700038761 |
| V3-5 | 514014184 | 700101793 |
| V3-5 | 516889361 | 700105334 |
| V3-5 | 517810313 | 700106930 |
| V3-5 | 533247696 | 700098131 |
| V3-5 | 553359145 | 700106542 |
| V3-5 | 604812005 | 700102318 |
| V3-5 | 604812005 | 700110438 |
| V3-5 | 612472597 | 700105181 |
| V3-5 | 638754422 | 700097928 |
| V3-5 | 638754422 | 700109252 |
| V3-5 | 643185023 | 700105496 |
| V3-5 | 650853796 | 700098888 |
| V3-5 | 650853796 | 700110375 |
| V3-5 | 663835652 | 700114246 |
| V3-5 | 668248235 | 700105904 |
| V3-5 | 675950834 | 700106259 |
| V3-5 | 682102541 | 700114872 |
| V3-5 | 686765762 | 700107208 |
| V3-5 | 686765762 | 700111045 |
| V3-5 | 706846339 | 700106170 |
| V3-5 | 737052003 | 700106093 |
| V3-5 | 763395383 | 700114017 |
| V3-5 | 763435843 | 700024470 |
| V3-5 | 763456073 | 700105723 |
| V3-5 | 763476303 | 700038520 |
| V3-5 | 763516763 | 700023023 |
| V3-5 | 763516763 | 700105856 |
| V3-5 | 763536994 | 700021876 |
| V3-5 | 763536994 | 700038472 |
| V3-5 | 763536994 | 700113616 |
| V3-5 | 763557224 | 700105459 |
| V3-5 | 763597684 | 700023313 |
| V3-5 | 763597684 | 700038053 |
| V3-5 | 763638144 | 700023226 |
| V3-5 | 763638144 | 700114287 |
| V3-5 | 763678604 | 700021824 |
| V3-5 | 763678604 | 700038158 |
| V3-5 | 763698834 | 700023096 |
| V3-5 | 763698834 | 700038740 |
| V3-5 | 763719065 | 700113541 |
| V3-5 | 763721271 | 700111318 |
| V3-5 | 763759525 | 700023845 |
| V3-5 | 763759525 | 700103710 |
| V3-5 | 763820215 | 700021934 |
| V3-5 | 763820215 | 700105238 |
| V3-5 | 763840445 | 700023267 |
| V3-5 | 763840445 | 700114125 |
| V3-5 | 763860675 | 700023578 |
| V3-5 | 763860675 | 700103478 |
| V3-5 | 763880905 | 700023633 |
| V3-5 | 763880905 | 700038578 |
| V3-5 | 763901136 | 700023788 |
| V3-5 | 763901136 | 700114653 |
| V3-5 | 763921366 | 700023477 |
| V3-5 | 763982056 | 700023936 |
| V3-5 | 763982056 | 700106647 |
| V3-5 | 764002286 | 700023539 |
| V3-5 | 764002286 | 700024509 |
| V3-5 | 764042746 | 700024044 |
| V3-5 | 764042746 | 700114480 |
| V3-5 | 764062976 | 700023654 |
| V3-5 | 764083206 | 700024107 |
| V3-5 | 764083206 | 700037560 |
| V3-5 | 764143897 | 700023902 |
| V3-5 | 764143897 | 700106837 |
| V3-5 | 764184357 | 700023987 |
| V3-5 | 764184357 | 700106056 |
| V3-5 | 764224817 | 700024437 |
| V3-5 | 764224817 | 700106695 |
| V3-5 | 764245047 | 700024173 |
| V3-5 | 764285508 | 700024233 |
| V3-5 | 764305738 | 700024566 |
| V3-5 | 764305738 | 700037484 |
| V3-5 | 764325968 | 700024615 |
| V3-5 | 764346198 | 700024254 |
| V3-5 | 764346198 | 700113562 |
| V3-5 | 764366428 | 700024809 |
| V3-5 | 764447348 | 700024673 |
| V3-5 | 764467579 | 700024379 |
| V3-5 | 764487809 | 700024318 |
| V3-5 | 764487809 | 700105372 |
| V3-5 | 764508039 | 700024711 |
| V3-5 | 764588959 | 700024752 |
| V3-5 | 764649650 | 700024866 |
| V3-5 | 764669880 | 700024930 |
| V3-5 | 764669880 | 700038263 |
| V3-5 | 764710340 | 700037026 |
| V3-5 | 764750800 | 700113035 |
| V3-5 | 764811490 | 700106914 |
| V3-5 | 764831721 | 700114749 |
| V3-5 | 764872181 | 700113093 |
| V3-5 | 764892411 | 700038386 |
| V3-5 | 764953101 | 700114419 |
| V3-5 | 765013792 | 700037200 |
| V3-5 | 765034022 | 700037738 |
| V3-5 | 765074482 | 700024998 |
| V3-5 | 765074482 | 700105612 |
| V3-5 | 765094712 | 700037074 |
| V3-5 | 765094712 | 700103653 |
| V3-5 | 765135172 | 700037284 |
| V3-5 | 765661155 | 700038322 |
| V3-5 | 765701615 | 700038414 |
| V3-5 | 809635352 | 700098451 |
| V3-5 | 809635352 | 700110834 |
| V3-5 | 840279516 | 700102678 |
| V3-5 | 857980665 | 700106784 |
| V3-5 | 861967750 | 700038902 |
| V3-5 | 863126187 | 700103575 |
| V3-5 | 866653606 | 700111244 |
| V3-5 | 872848689 | 700111767 |
| V3-5 | 892969023 | 700099978 |
| V3-5 | 892969023 | 700110309 |
| V3-5 | 901775393 | 700114767 |
| V3-5 | 937415040 | 700102541 |
| V3-5 | 937495960 | 700111596 |
| V3-5 | 953045535 | 700105632 |
| V3-5 | 964271349 | 700099400 |
| V3-5 | 970836795 | 700097994 |
| V3-5 | 970836795 | 700109129 |
