# Supplementary material for: Novel Bacterial Taxa in the Human Microbiome
Source: PLoS One. 2012 Jun 13;7(6):e35294. doi: 10.1371/journal.pone.0035294 (PMC3374617; doi:10.1371/journal.pone.0035294)
Supplement: Text S1 — Comparison of novel sequences from Roche WGS and variable region datasets. (DOC) [file pone.0035294.s007.doc]

**Text S1. Comparison of novel sequences from the Roche WGS and variable region datasets.**

In general, the RDP classification (Table S3), insertion in the Silva reference tree (Figure 3), OTUs (Table S3), and alignment results agree on which sequences are closely related. The RDP classifications correspond well to the OTU clustering. In each case where the RDP genus classification is not consistent within an OTU (OTUs 4, 7, 11–13, 26), those sequences that have a different genus from the majority of sequences in the OTU are classified by RDP with a very low bootstrap value (<0.44). With the exception of OTUs 7, 11, 25, and 26 and three sequences in OTUs 12 and 13 (discussed below), the placement on the Silva tree is also in line with the OTU classifications. The RDP classification at the phylum level agrees with the phylum-level placement on the Silva tree for all but two sequences (OTUs 9 and 22, discussed below), and in the cases where the novel sequence has a close sister taxon on the Silva tree, this usually agrees with the genus level RDP classification. Aligning the novel Roche WGS sequences to the novel Roche variable region sequences (using NCBI BLAST with default parameters) produces several alignments with 100% identity over the full length of the shorter of the two sequences. The alignment results are not expected to recapitulate all of the clustering and categorizing of the other methods since those methods leverage additional information, but the high-quality alignment results should be in agreement with the other methods. This is indeed the case, as all but one of the sequences which share high sequence similarity are from OTU 13 (the exception being from OTU 10 and discussed below). Furthermore, all but one sequence (SRX024348_SRR061731.319203) is classified as *Barnesiella*, and all the sequences are placed close together on the Silva tree. Discrepancies in the grouping of sequences depending on method are discussed below.

The first disagreement between the RDP classification at the phylum level and the placement on the Silva tree is for the single sequence in OTU 9. It is classified by RDP as the phylum Firmicutes but appears in the Tenericutes phylum in the Silva tree. However, the Tenericutes were recently split from the Firmicutes [35], so the discrepancies may be due to outdated taxonomy for one or more 16S references. The other discrepancy is for the single sequence in OTU 22, which is classified as Actinobacteria by RDP with a very low bootstrap value and is placed on the Firmicutes branch of the Silva tree. This is a short sequence (109 bp), and the first approximately 60 bp is a highly conserved region shared by the two phyla. The remaining portion of the sequence does not map well to any known taxon and thus the classification of this sequence is difficult, as reflected by the arbitrary phylum classification by different algorithms.

Disagreement between the placement of sequences in the Silva tree and the structure of the OTUs is due to the difficulty of identifying some of the novel sequences, as reflected by poor bootstrap values for the RDP classification. Sequences from OTUs 25 and 26 are not found on a single branch of the Silva tree and have poor RDP classification at the genus level. However, all the sequences in these OTUs are found within a small sub-tree of the Firmicutes tree, along with all the other OTUs classified to the family Ruminococcaceae (OTUs 3, 5, 6, 14, 21, and 23), confirming that these sequences are related at a higher taxonomic level. Sequences from OTUs 7 and 11 are spread more widely across the Firmicutes tree, but remain within the Clostridiales sub-tree, which contains OTUs 8, 16, 17, and 24, as well as the Ruminococcaceae sub-tree. The sequences in OTUs 7 and 11 are poorly classified even at the family level, reflecting that these sequences are even more difficult to classify. The exception is the two Roche V1-3 sequences in OTU 11 that are confidently assigned to the genus Dorea. These two sequences also appear close together on the Silva tree, along with a Dorea reference sequence.

The two largest OTUs, 12 and 13, are classified by RDP as the genus *Barnesiella* and the sequences from these OTUs are placed on three sister polytomic leaf nodes on the Silva tree. The leaf node with the largest number of leaves contains only novel sequences, while the other two leaf nodes each have novel sequence leaves as well as a reference sequence leaf from *Barnesiella*. In addition, three sequences from these OTUs are found slightly farther away on the Bacteroidetes sub-tree: sequence SRX024348_SRR061731.202172 is poorly classified by RDP at the family level as Marinilabiaceae, sequence SRX024348_SRR061731.319203 is poorly classified at the genus level as *Odoribacter*, and sequence SRX024348_SRR061731.488743 is poorly classified at the genus level as Paludibacter. The disagreement between methods here seems to indicate that these sequences are particularly difficult to classify. However, sequence SRX024348_SRR061731.319203 shares 100% sequence identity over 100% of its length with several other Roche V1–3 sequences from OTU 13 that were identified as *Barnesiella*, providing some evidence that the PhylOTU results hold merit.

The single-sequence OTUs 2, 10, and 15 are all identified by RDP as *Barnesiella* and are placed on the large polytomic leaf node with the sequences from OTU 12 and 13. The sequence in OTU 10 shares 100% sequence identity over 100% of its length with several sequences in OTU 13, indicating that PhylOTU may have spuriously split this sequence into its own OTU. In the case of OTUs 2 and 15, it is unclear if they were spuriously split from OTU 13 or if the tree analysis and the RDP genus level classification have insufficient resolution to distinguish true variation. In the case of OTU 15, the sequence is the only sequence identified as *Barnesiella* that is from the end of the 16S locus and thus shares no sequence overlap with other *Barnesiella* sequences, which may lead to difficulty in clustering it with other *Barnesiella* sequences.

Despite these minor variations between methods, the overall message remains clear. There is at least one (and likely at least two) new taxa related to the genus *Barnesiella* that have been observed in this dataset. The remaining sequences represent several distinct novel taxa. Although the correct clustering is difficult to ascertain with this data, at least 15 distinct novel taxa were observed.

35. Ludwig W, Schleifer KH, Whitman WB (2009) Revised road map to the phylum Firmicutes. Systematic Bacteriology 1-13.
